# Dentoalveolar changes and transverse discrepancies through the treatment of class II division 1 malocclusion in children using a twin block device

**DOI:** 10.4317/jced.62509

**Published:** 2025-03-01

**Authors:** Harun Achmad, Rasmidar Samad, Mansjur Nasir, Asmidar Anas, Sri Ramadhany, Arrang Sesioria

**Affiliations:** 1 Department of Pediatric Dentistry, Faculty of Dentistry, Hasanuddin University, Makassar, Indonesia; 2 Department of Public Health Education, Faculty of Dentistry, Hasanuddin University, Makassar, Indonesia; 3 Department of Orthodontic, Faculty of Dentistry, Hasanuddin University, Makassar, Indonesia; 4 Oral biology Department, Faculty of Dentistry, Hasanuddin University, Makassar, Indonesia; 5 Public Health Department, Faculty of Medicine, University Hasanuddin, Makassar

## Abstract

**Background:**

This study aims to analyze dentoalveolar changes and transverse discrepancies in the treatment of class II division 1 dentoskeletal malocclusion before and after treatment using a functional twin block device.

**Material and Methods:**

This study used a comparative analytical study to compare dentoalveolar changes and maxillary transverse discrepancies. The research sample was a study model of patients aged 7–15 years with 23 study models with class II division 1 dentoskeletal malocclusion cases at the clinic of the Department of Pediatric Dentistry, Hasanuddin University Educational Oral and Dental Hospital (RSGMP UNHAS). A sampling of the study model was carried out by purposive sampling. The measurement data was tested using the chi-squared normality test and then the statistical test was the paired t test.

**Results:**

Based on the statistical analysis of paired t-test there is a change in the distance between the mandibular canines, there is a change in the distance between the first premolars or between the mandibular first primary molars, there is a change in the distance between the second premolars or between the maxillary second primary molars, and there is a change in the distance between the maxillary molars after treatment using a twin block device.

**Conclusions:**

There were substantial dentoalveolar alterations in overjet, overbite, molar relation, and distance between premolars and molars after using the twin block device.

** Key words:**Malocclusion, twin block, dentoalveolar, jaw discrepancy.

## Introduction

A malocclusion is a form of maxillary and mandibular relationship that deviates from the standard form as a normal form (1). Malocclusion is associated with a less-than-ideal tooth relationship in a state of centric occlusion, which can lead to poor facial appearance, risk of caries and periodontal disease, and jaw joint disorders if not corrected (2,3). Malocclusion can occur in three directions, namely the sagittal, transverse, and vertical planes. Proper malocclusion treatment can be started at the optimal stage of maturation, which is at the peak of the growth spurt and will give the best results (4,5). Orthodontic treatment can only modify growth patterns, not produce growth. Determining the optimal timing of orthodontic treatment is closely related to the identification of growth spurts and thus can contribute significantly to the correction of skeletal disharmony (6-8).

Class II malocclusion is considered one of the most common craniofacial disorders. Class II malocclusion may be the result of a sagittal mandibular deficiency, maxillary excess or a combination of both (9,10). Class II malocclusions are abnormalities in the sagittal or anteroposterior plane that can be dental or skeletal (11). Increased overjet in any skeletal pattern can be caused by upper incisors that are proclinated and lower incisors that are retroclinated due to habit or soft tissues (12). Lips are generally inadequate in class II division 1, therefore they try to compensate with circumoral muscle activity, sliding the lower lip under the upper incisor, or a combination of all of these things (13,14). The development of this malocclusion may also be influenced by finger sucking or other oral habits, usually after imbalances in the buccinator muscles and tongue force, which narrow the maxillary arch. Additionally, habits often procline the top incisors while retrocline the lower incisors (15).

The occurrence of maxillary transverse discrepancy greatly affects many aspects, including the buccal-lingual inclination angle of the mandibular teeth (16). The role of the buccal-lingual inclination angle as compensation in the dental arch occurs to balance the misalignment, it occurs naturally and can change the formation of the arch length and buccal- lingual inclination angle of the mandibular teeth (17,18). The transverse discrepancy that occurs in the maxilla can cause a narrow maxillary palatal arch and is often accompanied by excessive vertical growth (19).

The midpalate suture is a growth area that contributes to the width of the maxillary arch, which is active until late adolescence. With increasing age, the suture will unite so that it will become more difficult to widen (20). Transverse plane discrepancy is a natural change of teeth that can be caused by local factors such as premature loss of primary teeth, agents and certain bad habits such as finger sucking or tongue thrusting (20,21). Transverse discrepancies can cause tooth asymmetry which basically involves shifting the median line, asymmetrical posterior tooth positions, asymmetrical arches, and divergent occlusal planes (21,22).

The development of class II appears to be promoted and maintained by both facial growth and dental eruption (23,24). Dentoalveolar changes can be seen through maxillary posterior segment distalization, maxillary incisor linguoversion, mandibular posterior segment mesialization and buccal tilt of mandibular incisors (24,25) Patients with an Angle class II malocclusion, which is characterized by an increased overjet and anterior crowding, molar distalization of the maxillary first permanent teeth may be an effective therapeutic option. Using intraoral or extraoral equipment, molar distalization can be accomplished (25,26).

Twin block devices are functional devices used for the early treatment of children with class II malocclusion by advancing the jaw and stimulating jaw growth. Twin block was developed by William J Clark in 1970 in Scotland and is currently one of the most common and most popular functional tools due to its effectiveness in correcting class II skeletal malocclusions (26,27).

Improvements in the dentoalveolar relationship in the sagittal direction can be measured through parameters such as decreasing overjet distance values, changes in overbite distance values, and changes in molar relation distances (28). Several observations of dentoalveolar changes resulting from treatment can also be seen in the combination of mandibular growth, distalization of upper molars and mesial-to-lower molar migration and proclination of lower incisors to labial resulting in decreased overjet (29). This study aimed to analyze dentoalveolar changes (overjet, overbite, and molar relation) and transverse discrepancies in the treatment of class II division 1 dentoskeletal malocclusion before and after treatment using a functional twin block device, based on measurements in a study model of pediatric patients of growth and development age.

## Material and Methods

This protocol was approved by the Health Research Ethics Committee for Dental and Oral Hospital, Faculty of Dentistry, Hasanuddin University, Ministry of Research, Technology and Higher Education, Indonesia (No.105/PL09/KEPK FKG-RSGM UNHAS/2022) on August 8, 2022. This research was conducted at the clinic of the Department of Pediatric Dentistry, Hasanuddin University Educational Oral and Dental Hospital (RSGMPUNHAS).

This research started from recruitment of participants on period March and June 2022. This research started from 10th August and 30th November 2022 and used a comparative analytical study to compare the results of measurements of maxillary dentoalveolar changes and transverse discrepancies in the patient study model. Sampling of the study model was carried out by purposive sampling. Each participant in this study was informed about the research and agreed and signed a written informed consent form to participate in this study. Screening was based on the inclusion criteria which were patients with dentoskeletal class II division 1, molar class II relationship and no previous orthodontic treatment. Subjects had to be included on prepubertal growth spurt phase. Exclusion criteria were patients who had not finished treatment during this study and patients who were not willing to participate in this study. The subject of this study was a study model of 23 patients (mean age 7-15 years) with class II dentoskeletal malocclusion treated with a twin block functional appliance. The study model used was a pre-treatment and post-treatment study model.

Assessment criteria in the study included dentoalveolar changes (overjet, overbite, and molar relation), dentoalveolar changes in the transverse plane consisting of changes in the distance between the canines, the distance between the first premolars or first primary molars, the distance between the second premolars or the second primary molars, the distance between the mandibular first permanent molars and the inclination of the mandibular first permanent molars before and after using the twin functional appliance.

Patient study models in good condition, with intact incisor tooth edges, canine tooth cusps, buccal premolar tooth cusps, and molar tooth central fossae, without any damage, are required. This study had exclusion criteria which included patient study models with no canines, permanent first molars, permanent first premolars or deciduous second molars and patient study models with partial loss of anterior teeth. Models that met the inclusion criteria were grouped into groups before and after treatment with a twin block device.

Intercanine width was measured by measuring the width of the intercanine width at the cusp apex of the right and left canines, interpremolar distance was measured between the buccal cusps of the right and left first premolars. The intermolar width was measured between the central fossa of the right and left molar teeth on the patient study model before and after treatment using a digital caliper in millimeters (mm) with an accuracy of 0.01. Measurements were taken thrice to minimize errors and ensure precise data analysis. Subsequently, the results were subjected to calibration, followed by data processing.

First, the data obtained were tested for normality using the Shapiro-Wilk test. The measurement data of the resulting research study models were then carried out using the chi-squared normality test to determine whether the research data is usually distributed and then determine the next statistical test in the form of a paired t-test. Analysis was done using RStudio 2022.07.1 Build 554 (RRID: SCR_000432).

## Results

- Dentoalveolar changes (overjet, overbite, and molar relation)

The results of the normality test on the overjet, overbite and molar relations variables using the Shapiro-Wilk test stated that the measurement results of the data produced in this study were normally distributed ( Table 1).

Data analysis was continued using a paired t-test to compare the results of the maxillary transverse dimension measurements on the research model before and after treatment using the twin block functional tool as shown in  Table 2.

a. Different test due to overjet impact

**Figure 1:**
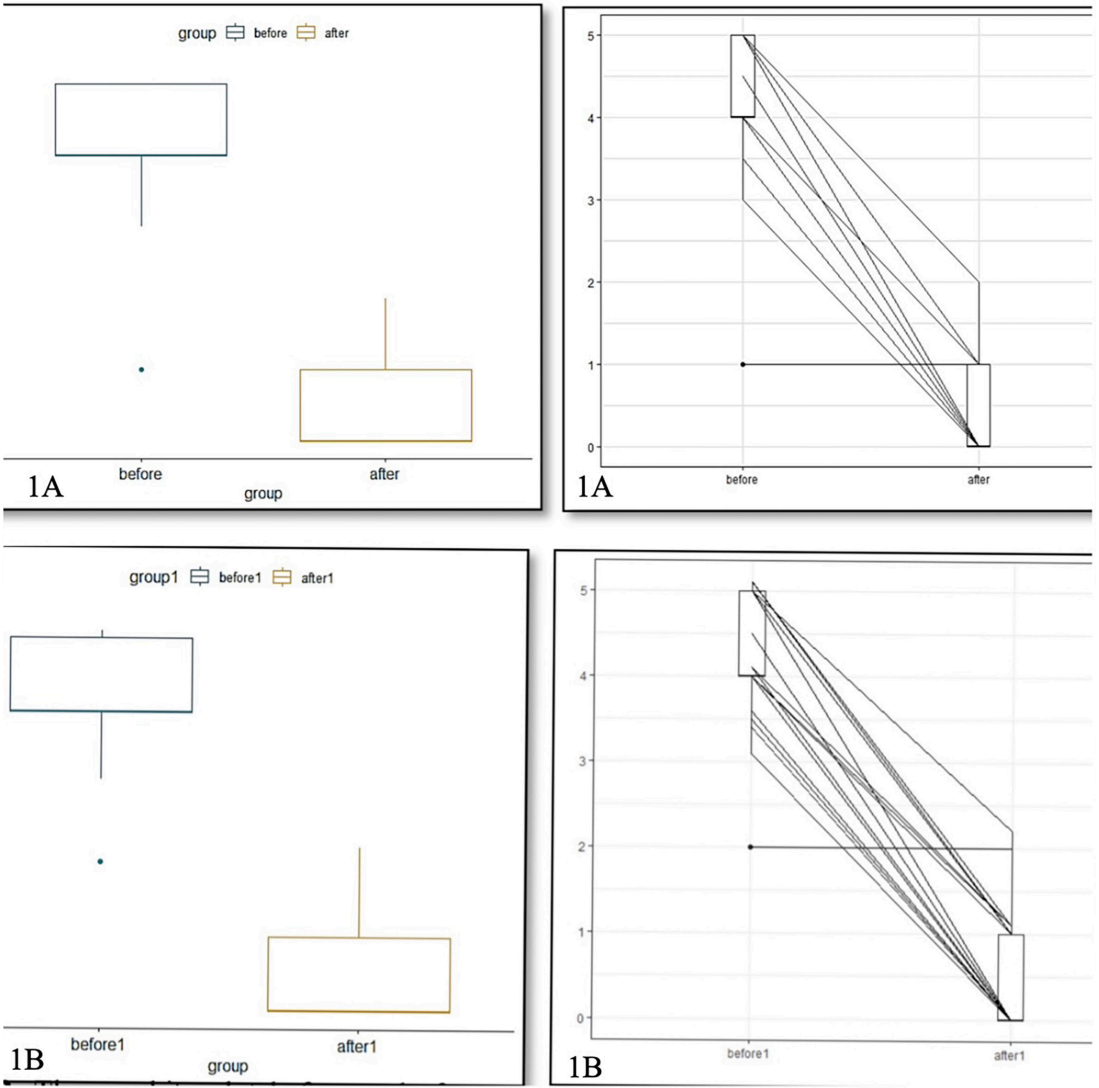
A,B) The overjet plot before and after being given treatment with twin block devices concluded that the conditions before and after treatment had a significant difference, indicated by the rejection of the null hypothesis. If the p-value < alpha, then reject H0. With a confidence level of 95 percent, the alpha tolerance level is 5 percent or 0.05, it is said to reject H0 because the p value is 7.17 x 10-13 which is less than the 5% alpha value. The average difference for each observation is 3.619408.

In Figure 1A, the average overjet value after being given treatment with a twin block device is smaller than before being given treatment. This can be seen from the range of values before and after treatment is given according to Figure 1B. Based on the average difference test (paired test), it can be concluded that the conditions before and after treatment with twin block devices have significant differences.

b. Different tests due to the impact of overbite

According to data in Figure 1B, the mean overbite value post-treatment is lower than the overbite value pre-treatment with the twin block appliance. Based on the paired t-test, it can be concluded that the conditions before and after treatment with twin block tools have significant differences.

c. Difference test due to the impact of molar relations

The average value of the molar relation distance after being given treatment with a twin block device is smaller than before being given treatment using a twin block device. This can be seen from the range of values before and after being given treatment for twin block devices. Based on the average difference test (paired test), it can be concluded that the conditions before and after have a significant difference.

- Dentoalveolar changes in the transverse plane

The results of the paired t-test calculation are presented in  Table 3, showing the mean value of the treatment on dentoalveolar changes as seen through the distance between canines, the distance between first premolars or first molars, the distance between second premolars or second molars and the distance between molars after treatment is greater than before treatment. This means that the functional twin block device produces dentoalveolar changes in the transverse plane. The largest change was seen in the mean distance between molar teeth (M) which increased by 3.12 mm (SD+2.49), and the smallest was seen in the mean canine tooth distance (C) which increased by 1.6 mm (SD+1.43).

**Figure 2:**
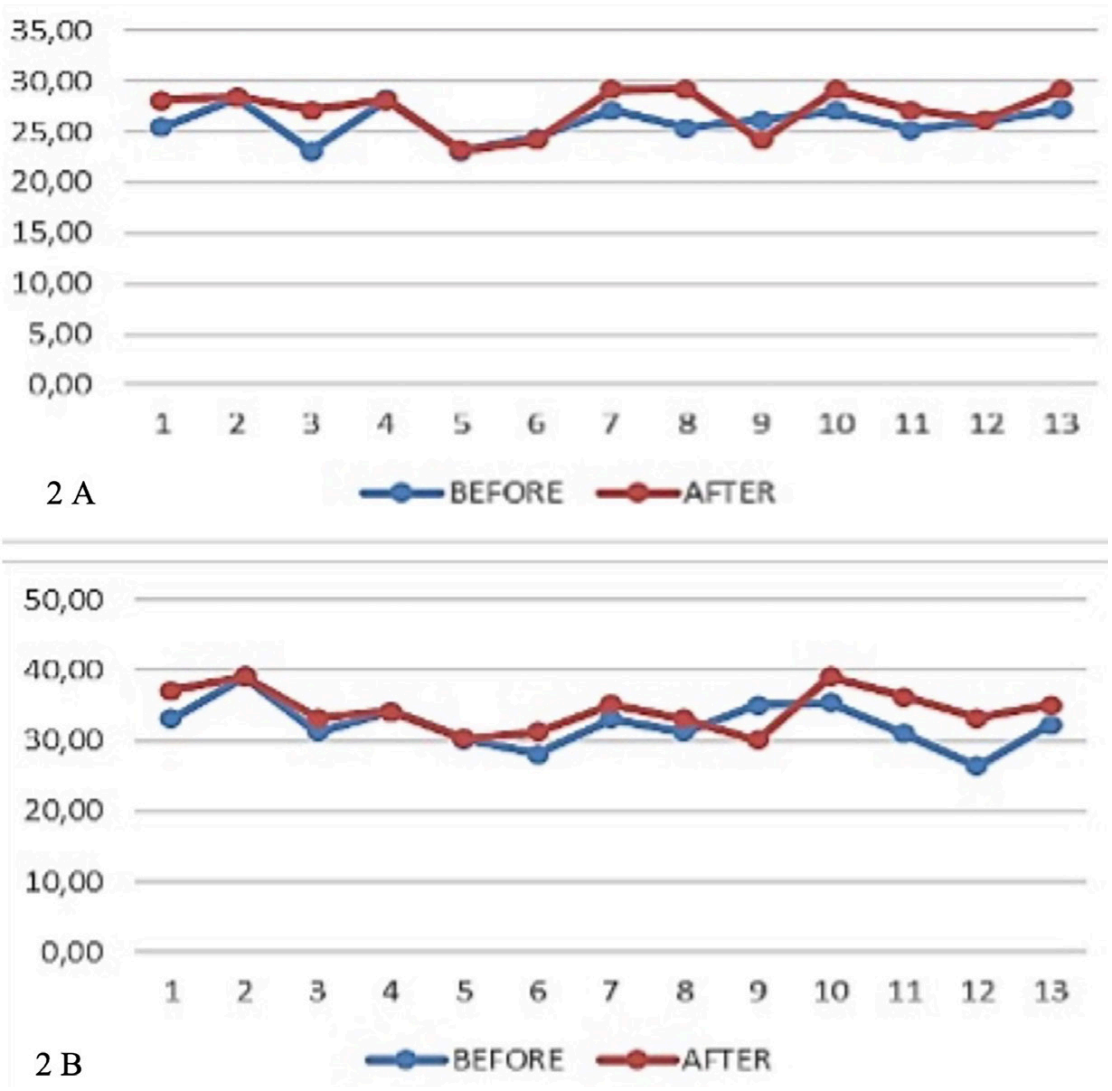
A) The results of the analysis with the paired t-test showed a P value of 0.026, this means that there is a significant (meaningful) difference between the average C parameter values before and after treatment. The average C parameter before treatment was 25.9177 ± 1.71455 and the average C parameter after treatment was 27.1677 ± 2.13794. B) The results of the analysis using the paired t-test showed a P value of 0.026, this means that there is a significant (meaningful) difference between the average P/M1parameter values before and after treatment. The average P/M1 parameter before treatment was 32.3515 ± 3.29067 and the average P/M1 parameter after treatment was 34.4138 ± 2.97241.

The dentoalveolar changes in the transverse plane in the value of the distance between the canines, before and after using the twin block functional device are shown in Figure 2A.

Transverse plane dentoalveolar changes in the value of distance between first premolars (mm) or first primary molars (P/M1) (mm), before and after using the twin block functional device as shown in Figure 2B.

**Figure 3:**
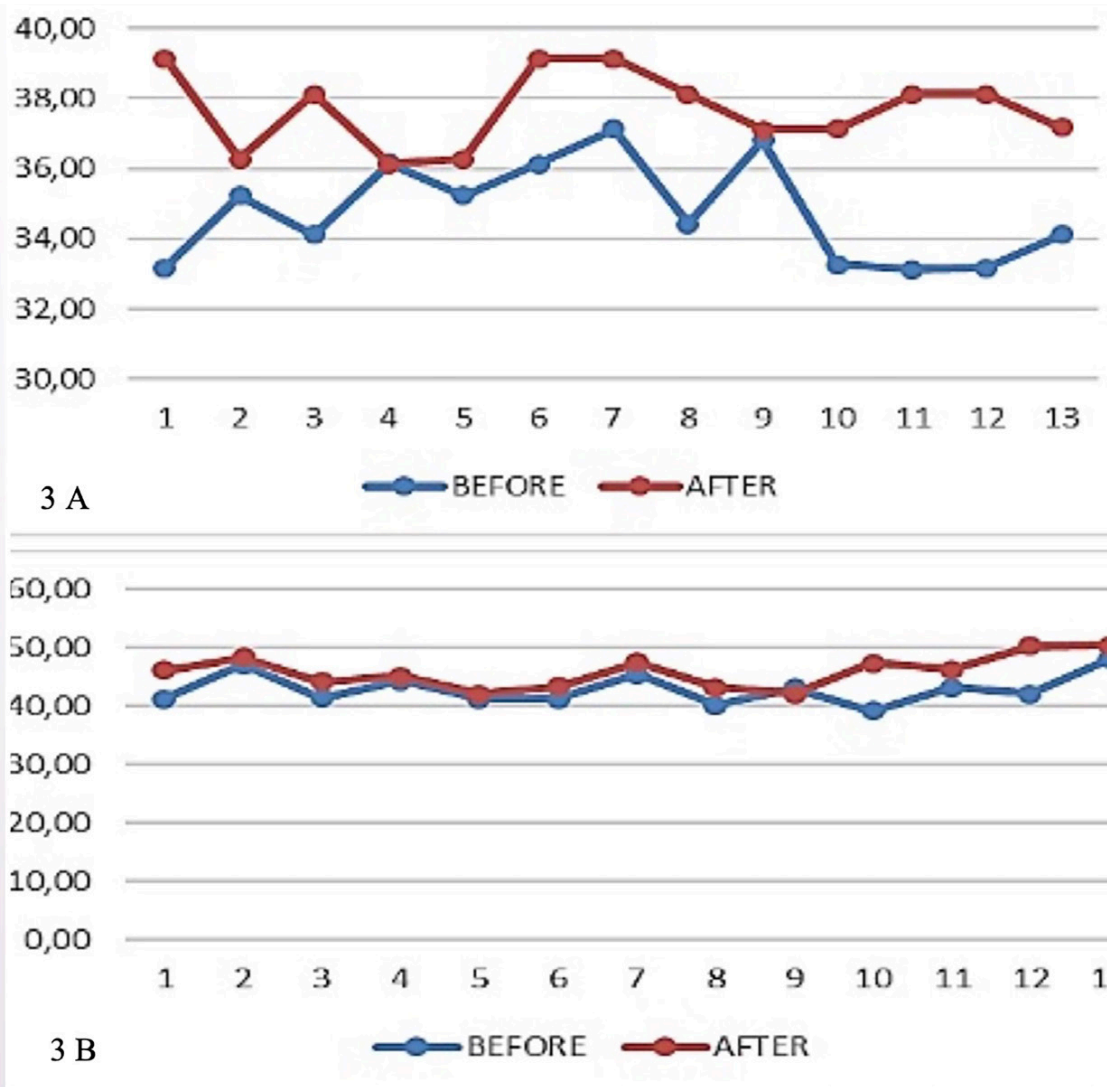
A) The results of the analysis with the paired t-test showed a P value of 0.000, this means that there is a significant difference (meaningful) between the average P/M2 parameter values before and after treatment. The average P/M2 parameter before treatment was 34.7785 ± 1.44097 and the average P/M2 parameter after treatment was 37.6908 ± 1.09521. B) The results of the analysis with the paired t-test showed a P value of 0.002, this means that there is a significant difference (meaningful) between the average M parameter values before and after treatment. The average parameter M before treatment was 42.9077 ± 2.65714 and the average parameter M after treatment was 45.8731 ± 2.82893.

Transverse plane dentoalveolar changes at the rate of the distance between the second premolars (mm) or the second primary molars (P/M2) (mm), before and after using the twin block functional device as shown in Figure 3A.

Transverse plane dentoalveolar changes in the value of distance between molars (M) (mm), before and after using the twin block functional device as shown in Figure 3B.

Data from the research results of calculating the distance between canines, between mandibular first premolars or mandibular first molars, distance between second premolars or mandibular first molars and distance between mandibular first permanent molars using the paired T test showed a *p* value <0.05 so it can be concluded that there are differences in the distance between canines, distance between first premolars or first permanent molars ( Table 3).

**Figure 4:**
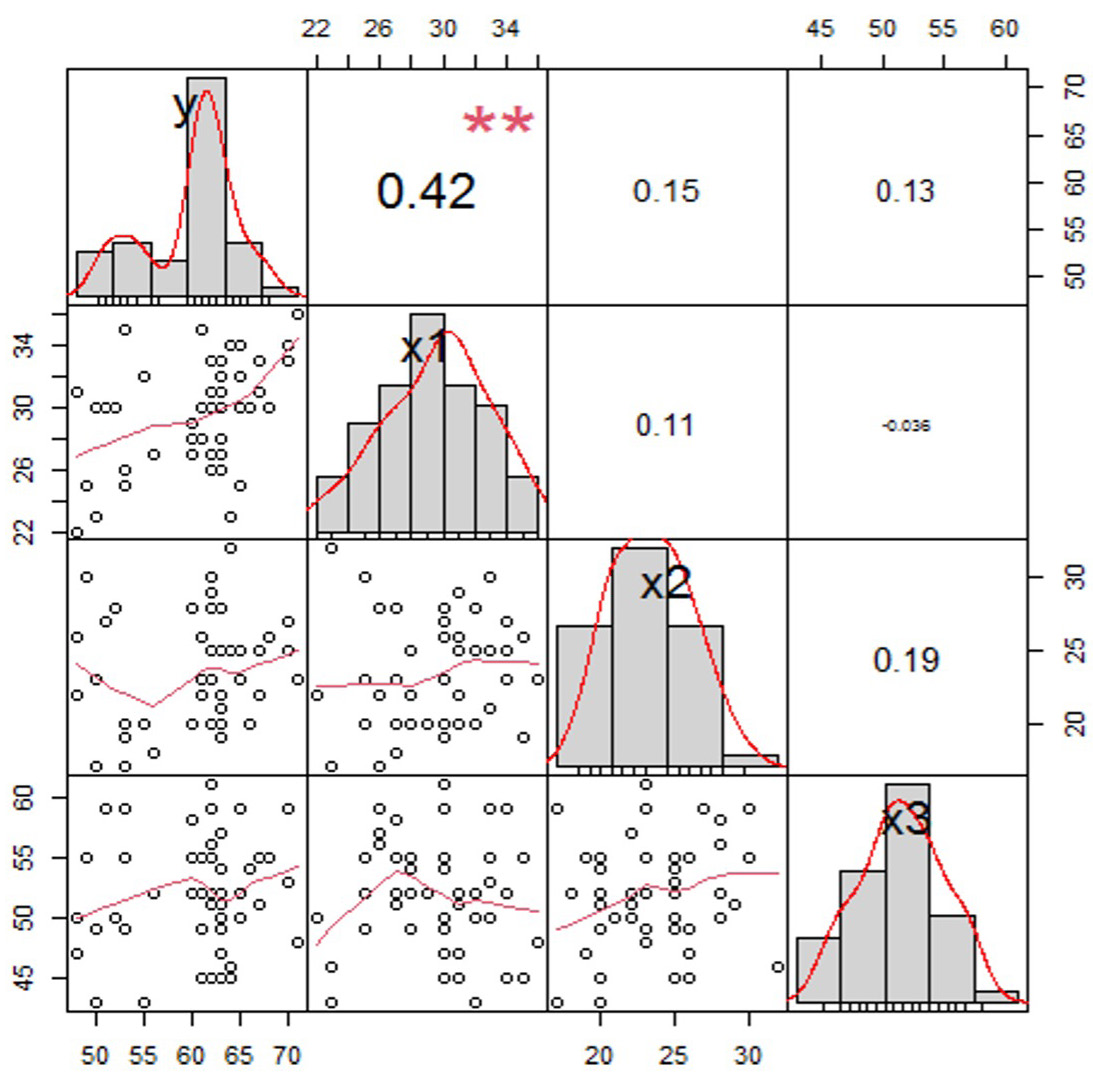
Variable correlation matrix Y, X1, X2 and X3.

Based on the identification and analysis of  Table 3, the linear variables of lower anterior facial height (Y), maxillary anterior dentoalveolar height (X1), maxillary posterior dentoalveolar height (X2), and maxillary length (X3) have a close relationship. This identification can be analyzed from Figure 4 that based on the diagonal side of the correlation matrix, shows the distribution of data on the variables used. When viewed from the Y, X1 and X3 variables where the distribution tends to be negative or tends to the left, it can be interpreted that the calculated average value is smaller than the median and mode values, which means that the data tends to cluster at high values.

However, it is different from the X2 variable whose data distribution tends to be positive or tends to the right, which means the data tends to group into lower values. Judging from the level of sharpness of the graph, there are no outliers in the data. Viewed from the right side of the correlation matrix, the correlation between variables Y and X1 is stronger than X2 and X3 with variable Y, this is evidenced by the correlation value between variables y and x1 by 42 percent while X2 and X3 have a correlation of 0.15 each and 0.13 with Y variable.

## Discussion

Class II division I malocclusion is the most common class II condition. One alternative treatment for class II malocclusion is the use of a functional twin block device (30). The purpose of using a twin block device is to stimulate mandibular growth through growth stimulation of the condylar cartilage and functionally limiting maxillary growth (22). The effectiveness of the twin block device for the treatment of class II division 1 malocclusion can be assessed from the presence of dental and skeletal changes (31,32). The advantages of functional therapy are in the correction of the molar relation, the total length of the mandible and the height of the ramus are significantly increased and the direction of growth of the condyle is more posterior (31).

The main changes caused by functional appliances to the dentoalveolar include distalization of the maxillary posterior segment, lingual displacement of the maxillary incisors, mesialization of the mandibular posterior segment and buccal inclination of the mandibular incisors resulting in changes in overjet, overbite and changes in molar relationships (33). Treatment of class II division 1 malocclusion with functional fixed appliances will show maximum results if performed at the optimal skeletal maturation stage (34).

The results of the study in  Table 2 show the three variables of dentoalveolar changes before and after treatment using twin blocks in 23 samples of the studied study model. The difference in the decrease in the value of overjet, overbite and molar relations is caused by several factors, including patient compliance, operator skill, duration of use, patient age, race and ethnicity of the patient and the initial clinical condition of the patient (35). The patient’s large overjet and overbite at the start of treatment were due to lack of mandibular growth (36). Patients with slower jaw growth tend to be stimulated more quickly with functional devices because of the influence of growth hormone in early jaw growth (37,38).

The effectiveness of treatment with a twin block device can be assessed from the decrease in the value of overjet, overbite, and changes in the molar relationship to class 1 Angle which is presented in  Table 2 (39). All samples studied showed significant changes in all variables of the value of overjet, overbite, and molar relations. The variable overjet value decreased by an average of 5.09 mm from an average before treatment of 9.02 mm to 3.93 mm after treatment. This happens because the twin block device functions to stimulate mandibular

growth through growth stimulation of the condylar cartilage, so that endochondral growth will occur more rapidly than it should (39,40). The twin block device is also designed to advance the patient’s lower jaw so that it inhibits maxillary growth and stimulates anterior mandibular growth thereby reducing the overjet value (39,41).

The same thing also happened to the overbite value variable, which experienced a decrease in average of 2.17 mm from the mean before treatment of 4.53 mm to 2.36 mm after treatment. This is due to the increase in mandibular body length and ramus height. The twin block device also stimulates vertical growth causing intrusion of the posterior teeth, while the anterior teeth remain free to erupt, this effect helps to increase the overbite and bring the anterior teeth into occlusion (42).

The variable value of the molar relation decreased by 3.82 mm on average from 4.28 mm before treatment to 0.46 mm after treatment. This happens because the twin block functional device works to stimulate the mandible to grow and move forward to improve the molar relationship which was previously class II Angle to class I Angle. The use of a twin block device also causes the mandible to move forward which depends on the activity of the tip of the condyle to carry out the profiling of the diseased tissue that is converted into bone. In addition, the processes of apposition and resorption of the anterior and posterior portions of the ramus and elongation of the posterior teeth are also responsible for the advancement of the mandible. The advancement of the mandible causes a change in the ideal molar relation to be achieved (43).

The results of this study are in line with research conducted by Al Anezi *et al*., Singh *et al*., and Fareen *et al*. who stated that the twin block device will reduce the value of overjet, overbite and improve the molar relationship from class II to class I Angle (44). According to Bacheti in his research, the most visible changes after twin block treatment were dentoalveolar changes. The success of the treatment of class II division 1 dentoskeletal malocclusion is highly dependent on the patient’s cooperative attitude, in addition to other factors such as growth patterns, etiology and severity of the initial malocclusion (44-46).

Evaluation of dentoalveolar changes in the transverse plane can be seen from the distance between the canines, the distance between the first and second premolars or the first and second primary molars, the distance between the molars and changes in the inclination of the mandibular molars. The functional twin block device was used to keep the posterior teeth straight and resulted in an increase in the length of the anterior arch (47,48).

Based on the results of the paired t-test in calculating changes in the distance between the canines, it was found that the treatment using twin block devices can produce significant changes in changes in the distance between the canines (49). This occurs because the twin block device uses an expansion coupler, an active device that generates power in the transverse direction, and because the use of twin block devices will cause changes in the spacing between the canines following treatment with an expansion device on the jaw with noticeably more stable outcomes (49,50).

Treatment using a twin block device is quite effective for adjusting the distance between canines, where this study shows that the canine distance changes on average by 1.61 mm (SD 1.43). Motoyoshi in 2005 stated that changes in the value of the distance between canines at the age of 6 to 11 years can occur due to mandibular growth (37). There were also significant changes in the distance between mandibular first premolars or first molars. The movement due to expansion in the premolar region is relatively more stable than the canines and molars. BeGole’s study stated that during expansion, the canines experienced less expansion than the premolars. Swadowsky and Lauderdale stated that the best level of stability after mandibular expansion treatment was in the second and first premolars (31).

The average change in the distance between the molars was 3.12 mm (SD 2.49) measured from the mesial cusp buccal point. This study is in line with the results of Motoyoshi *et al*. 50 which stated that the average mandibular molar distance increased by 5.42 mm after the use of twin block devices for 6 months to 1 year. The conclusion is that there are expansion forces on the skeletal and dentoalveolar with the twin block appliance.

Variable changes in molar inclination in treatment using twin block devices experienced a reduction in the angle so that the molar inclination became more upright. The use of the twin block device resulted in a change in the inclination to the buccal direction so that the molar inclination became upright, which previously had a maxillary transverse discrepancy. Dentoalveolar changes through examination of the study model are easier to carryout, applicable and eliminate the resulting impact than radiation examination, but have a disadvantage, namely that the study model print may be damaged in the part to be measured, making it difficult to make measurements.

## Conclusions

The study was conducted using a patient study model to look at two things, namely dentoalveolar changes and in the transverse discrepancy of the jaw before and after using the twin block device. Dentoalveolar changes found were significant differences in overjet, overbite and molar relations before and after treatment with twin block devices. There was a change in the distance between the second premolars or between the maxillary second primary molars after treatment using a twin block device. There was a change in the distance between the maxillary molars after treatment using a twin block device.

## Figures and Tables

**Table 1:** Normality test of dentoalveolar changes variable.

Average (SD) mm			
	Before	After	Difference
Overjet value	8.22 (2.351)	3.53 (1.155)	4.69 (1.325)
Overbite value	5.53 (0.850)	1.41 (0.849)	4.12 (0.778)
Molar relation	5.35 (0.831)	1.46 (0.717)	3.89 (0.663)

**Table 2:** Results of paired t-test of dentoalveolar changes variable.

Variable		Average	Standard Deviation	Chi-square	
				p-value	description
Molar relation	Before	5.35	0.831	0.8456*	significant
	After	1.46	0.717	0.4456*	significant
	Difference	3.89	0.663	0.3524*	significant
Overjet value	Before	8.22	2,351	0.8453*	significant
	After	3.53	1.155	0.4466*	significant
	Difference	4.69	1.325	0.2850*	significant
Overbite value	Before	5.53	0.850	0.9835*	significant
	After	1.41	0.849	0.8825*	significant
	Difference	4.12	0.778	0.0738*	significant

**Table 3:** Statistical results of paired t-test comparing changes in dentoalveolar distance in the transverse plane.

	Average ± SD			
	Before	After	Difference	p-value
Value of distance between canines (C) mm	25.92 ± 1.72	27.17±2.14	1.61±1.43	0.00050*
Value of distance between first premolars (mm) or first primary molars (P/M1) (mm)	32.35 ± 3.29	34.42 ± 2.97	2.85±2.12	0.00011*
Value of distance between second premolars (mm) or second primary molars (P/M2) (mm)	34.78 ± 1.44	37.69 ± 1.10	2.92±1.91	0.00003*
Value of distance between molars (M) (mm)	42.91 ± 2.66	45.87±2.83	3.12±2.4	0.00021*

## Data Availability

Underlying data Open Science Framework: Dentoalveolar changes and transverse discrepancies through the treatment of class II division 1 malocclusion in children using a twin block device. https://doi.org/10.17605/OSF.IO/9JTUE76 This project contains data of dentoalveolar changes through measuring overjet, overbite and intermolar relations using the difference test before and after treatment with the twinblock appliance • Data File 1: Linear Measurement Results of Lower Anterior Face Height, anterior maxilary Dentoalveolar Height, Maxillary Posterior Dentoalveolar Height and Maxillary Length .pdf • Data File 2 : Analysis of dentoalveolar changes in overbite, overjet and intermolar before and after twinblock treatment. pdf • Data File 3: Dentoalveolar changes test result overbite, overjet and intermolar before and after twinblock treatment. pdf
